# Safe and Effective Reduction Technique for Intertrochanteric Fracture with Ipsilateral Below-Knee Amputated Limb

**DOI:** 10.1155/2017/2672905

**Published:** 2017-05-14

**Authors:** Hironori Ochi, Tomonori Baba, Takahiro Hamanaka, Yu Ozaki, Taiji Watari, Yasuhiro Homma, Mikio Matsumoto, Kazuo Kaneko

**Affiliations:** Department of Orthopedic Surgery, Juntendo University School of Medicine, Tokyo, Japan

## Abstract

The positioning of the patient on the fracture table is critical for the successful reduction and operative fixation of intertrochanteric hip fractures. However, this manipulation is challenging with patients who have undergone amputations of their legs. A 97-year-old man presented to the emergency department with symptom of right hip pain following a mechanical fall. He had a below-knee amputation on his right leg following a traffic accident as a 19-year-old and had a below-knee patellar tendon bearing prosthesis fitted to his lower limb for mobility. Radiographs of his pelvis revealed a displaced intertrochanteric fracture of the right side femur. The patient was positioned on a fracture table, as in the standard procedure. The method of inverting the traction boot to accommodate the flexed knee and stump described by Al-Harthy could be used to provide traction and rotational control. Internal fixation was performed using a short femoral nail. Postoperatively, the patient could walk with full weight bearing using a prosthesis on his affected limb. The method of inverting the traction boot to accommodate the flexed knee and stump can be used safely and effectively to achieve and maintain fracture reduction during fixation of intertrochanteric fractures for patients with a below-knee amputated limb.

## 1. Introduction

Fractures of the neck and intertrochanteric region of the femur are common worldwide [1, 2]. Intertrochanteric hip fractures are generally treated by closed reduction and internal fixation with a dynamic hip screw or an intramedullary device using a fracture table [1, 3]. The positioning of the patient on the fracture table is critical for the successful reduction and operative fixation of the fracture [4]. This involves applying traction and rotation on the legs, after placing the feet in boots fixed to the table [5]. However, this manipulation is challenging with patients who have undergone amputations of their legs. A few methods have been described for patients with a below-knee amputation undergoing fixation for intertrochanteric fractures [3, 6, 7]. We describe in detail a safe and effective technique to overcome this problem for a patient with below-knee amputation.

## 2. Case Report

**Figure 1:**
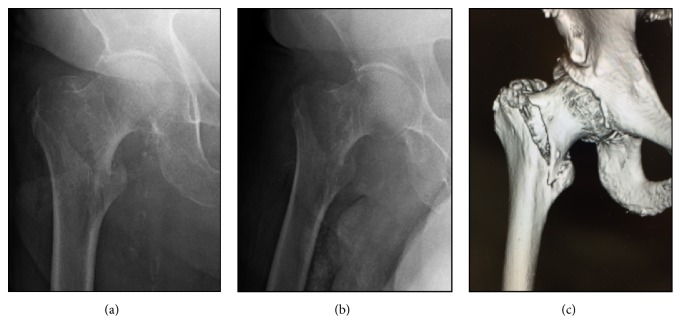
Preoperative anteroposterior (a) and lateral (b) radiographs of the right hip demonstrating a displaced intertrochanteric femoral fracture. Three-dimensional computed tomography (c) also demonstrated a displaced intertrochanteric femoral fracture.

A 97-year-old man presented to the emergency department at our hospital with symptom of right hip pain following a mechanical fall. He had a below-knee amputation on his right leg following a traffic accident as a 19-year-old and had a below-knee patellar tendon bearing prosthesis fitted to his lower limb for mobility. He could walk using a T-cane with the prosthesis. Radiographs and computed tomography of his pelvis revealed a displaced intertrochanteric fracture of the right side femur (AO classification: 31-A1) (Figure 1). Internal fixation of the fracture using an intramedullary device was planned and the patient provided informed consent.

**Figure 2:**
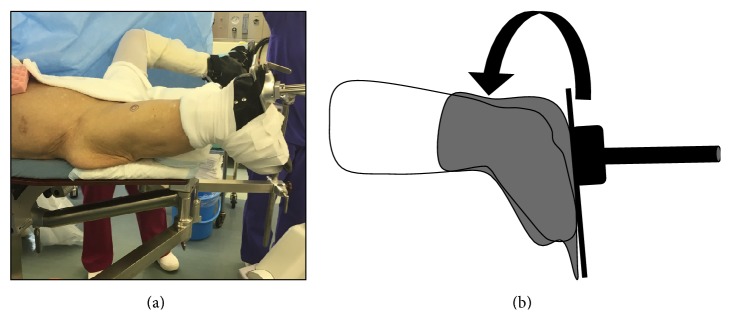
Intraoperative photographs demonstrating the setup of the patient on the fracture table with the amputated limb secured with the boot inverted to accommodate the flexed knee and stump (a). Schema of this method (b).

**Figure 3:**
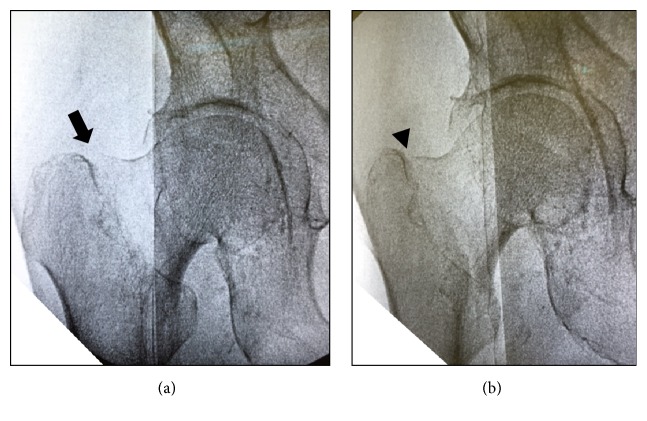
(a) The fracture was displaced to be shortening and varus (arrow). (b) After the reduction technique with traction and internal rotation, the displaced fracture was reduced (arrowhead).

**Figure 4:**
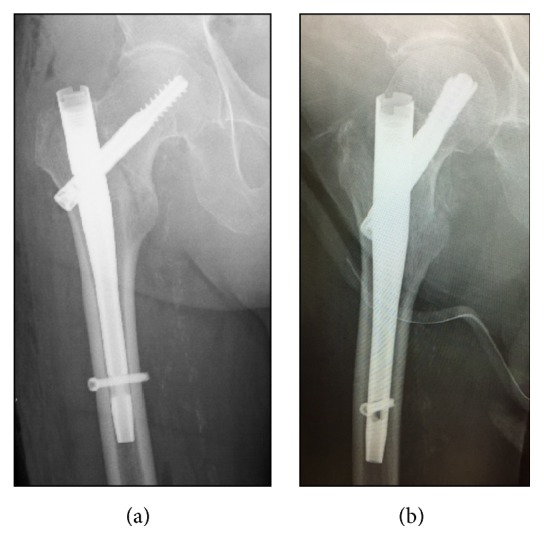
Postoperative anteroposterior (a) and lateral (b) radiographs of the pelvis demonstrating satisfactory short femoral nail fixation of the intertrochanteric hip fracture.

**Figure 5:**
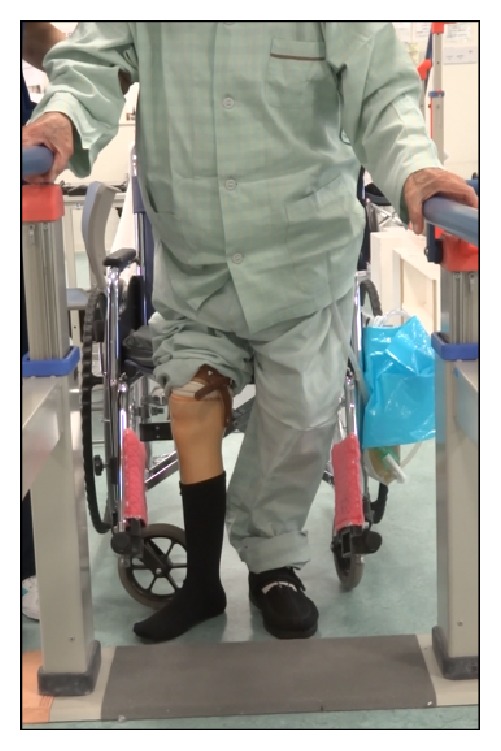
The patient could walk with full weight bearing with a prosthesis on his right affected limb after the operation.

The patient was positioned on a fracture table, as in the standard procedure. The method of inverting the traction boot to accommodate the flexed knee and stump could be used to provide traction and rotational control (Figure 2). Reduction manipulation was performed. First, a sufficient amount of traction and then internal rotation were done (Figure 3). Internal fixation was performed using a short femoral nail (U-Blade Lag Screw for the Gamma3® nail: Stryker Orthopaedics) (Figure 4). There were no complications including skin injury or infection in the affected limbs intraoperatively and postoperatively. Postoperatively, rehabilitation was started on the next day and the patient could walk with full weight bearing using a prosthesis on his affected limb (Figure 5).

## 3. Discussion

Patients with below-knee amputations and intertrochanteric fractures on the ipsilateral side have a characteristic problem; positioning their legs on the fracture table is difficult because of the absence of the foot. This problem is accentuated when reduction is needed for a more displaced fracture [3]. In our case, the method of inverting the traction boot to accommodate the flexed knee and stump described by Al-Harthy could be used safely and effectively to achieve and maintain fracture reduction during fixation of the intertrochanteric fracture for our patient with a below-knee amputated limb (Figure 2) [6]. This method has the advantage that at the time of reduction of rotation we could control the affected limbs with the patella as a guide. We also thought that this improvement in manipulating the affected leg would be an advantage when selecting implants for internal fixation.

Several methods of supporting the fractured limb on the traction table have been described in patients with intertrochanteric fractures and below-knee amputations (Table 1) [3, 6–8]. Each method should be considered based on its advantages and disadvantages (Table 1). (1) The first method is inverting traction boot [6]: inverted traction boot to accommodate the below-knee stump with the knee flexed attached to the traction table. The advantages of this method were that the reduction manipulation and maintaining fracture reduction were better and the risks of skin injury and infection were lower. In particular, at the time of reduction of rotation control, we can check the rotation of the affected leg using the patella as a guide. However, this method has a limitation about the length of stump (the stump should be 12 cm or more) [3]. This method was considered optimal in our case, so we checked whether the inverted traction boot could accommodate the below-knee stump preoperatively. (2) The second method is skin traction [7]: using strapping and elastic bandages attached to the traction table. Although the risks of skin injury and infection and influence of stump length were lower, the reduction manipulation and maintenance of fracture reduction were difficult. (3) The third method is skeletal traction [8]: using a Steinmann pin with hoop or external fixator attached to the traction table. Although this method provides sufficient traction, is gentle on the skin, and is less influenced by stump length, this method does not provide stable rotational control and it is associated with risks of infection, cutting out of the pin, and chronic skin scar discomfort. (4) The fourth method is manual traction [3]: manual reduction and maintenance of fracture reduction by a human assistant. Although this method is gentle on the skin and is less influenced by stump length, it does not provide sufficient traction and rotational control. Furthermore, it is very difficult to maintain fracture reduction during operation.

The method of inverting the traction boot to accommodate the flexed knee and stump can be used safely and effectively to achieve and maintain fracture reduction during fixation of intertrochanteric fractures for patients with a below-knee amputated limb. Each method should be considered based on its advantages and disadvantages for the patient condition and the type of fracture.

## Figures and Tables

**Table 1:** Advantages and disadvantages of methods from previous reports.

	Reduction		Maintaining reduction	Risk of skin injury	Risk of infection	Influence of long stump
	Traction	Rotation				
Inverting boot traction [6]	A	A	A	A	A	B
Skin traction [7]	B	C	B	A	A	A
Skeletal traction [8]	A	B	A	B	C	A
Assistant traction [3]	B	B	C	A	A	A

Several methods from previous reports of supporting the fractured limb on the traction table in patients with intertrochanteric fracture and below-knee amputations. Advantages and disadvantages of each method are shown as an assessment grade. Assessment grade: A (good), B (fair), and C (poor).
